# Effects of Long-Term Nutrient Input on Progeny Seed Nutrient Contents, Germination and Early Growth Characteristics of Typical Coastal Wetland Plants

**DOI:** 10.3390/plants14213393

**Published:** 2025-11-05

**Authors:** Rong Hu, Sifan Peng, Bo Guan, Hongxiang Zhang, Fanzhu Qu, Xuehong Wang, Zhikang Wang, Jisong Yang, Feilong Huang, Guangmei Wang, Guangxuan Han

**Affiliations:** 1 The Institute for Advanced Study of Coastal Ecology, Ludong University, Yantai 264025, China; 2 Key Laboratory of Wetland Ecology and Environment, Northeast Institute of Geography and Agroecology, Chinese Academy of Sciences, Changchun 130102, China; zhanghongxiang@iga.ac.cn; 3 Key Laboratory of Coastal Environmental Processes and Ecological Remediation, Yantai Institute of Coastal Zone Research, Chinese Academy of Sciences, Yantai 264003, China; 4 Yellow River Delta Field Observation and Research Station of Coastal Wetland Ecosystem, Chinese Academy of Sciences, Dongying 257500, China

**Keywords:** *Suaeda salsa*, *Phragmites australis*, N and P supply, seed nutrient content, seed germination

## Abstract

Seed reproduction is a vital stage in the life cycle of plants. In coastal wetlands, where nutrient regimes are increasingly altered by climate change and anthropogenic inputs, understanding how long-term nutrient enrichment affects progeny seed germination and early seedling establishment is essential for predicting vegetation shifts. In this study, we conducted a long-term nutrient addition experiment (2015–2024) in the Yellow River Delta with three nitrogen (N) and phosphorus (P) supply levels (Low, Medium, and High) and three N:P ratios (5:1, 15:1, and 45:1) to examine the effects of nutrient enrichment on seed nutrient contents, germination, and early seedling growth characteristics of *Suaeda salsa* and *Phragmites australis* measured in late 2024. Results showed that long-term fertilization increased the P content in *S. salsa* seeds by 17.1% to 146.0%, whereas N content was less influenced. In contrast, both N and P contents of *P. australis* seeds remained relatively stable across treatments. The seed germination and early seedling growth of the two species responded differently to various fertilization treatments. *S. salsa* seeds showed higher sensitivity to long-term fertilization, with improved behaviors under high nutrient level or 5:1 supply ratio. In contrast, *P. australis* performed better under medium nutrient level or higher N:P ratios (15:1 or 45:1). Correlation analysis indicated that P content in *S. salsa* seeds was significantly positively correlated with germination percentage, germination rate, germination index, and early seedling biomass, while N content was significantly correlated only with biomass. No significant correlations were observed between seed nutrients and germination or early seedling growth in *P. australis*. These findings underscore species-specific strategies in response to long-term nutrient enrichment and may ultimately influence species coexistence, community succession, and the resilience of coastal wetlands under ongoing global change.

## 1. Introduction

Coastal wetlands, situated at the ecotone between terrestrial and marine ecosystems, represent a critical wetland type characterized by complex ecological functions and significant ecological value. Their unique geographical position enables them to play pivotal roles in water purification, nutrient regulation, biodiversity conservation, and carbon sequestration [1,2]. In recent decades, intensified anthropogenic activities, particularly in coastal regions, have led to substantial inputs of nutrients such as nitrogen (N) and phosphorus (P) into natural ecosystems, resulting in increasingly severe wetland eutrophication [3]. Furthermore, climate change has emerged as another critical factor influencing ecological processes in coastal wetlands. Global warming, increased frequency of extreme precipitation events, and sea-level rise are exacerbating the complexity of wetland nutrient cycling by altering hydrological dynamics [4], salinity gradients [5], and plant–microbe interactions [6]. Through complex biogeochemical processes including sediment deposition, plant nutrient uptake, and microbial transformation of N and P, coastal wetlands serve irreplaceable functions in mitigating exogenous nutrient inputs and maintaining aquatic nutrient balance [7]. Although natural coastal wetlands occupy merely 0.5% of global land area, their ecological contributions to N removal and blue carbon sequestration are disproportionately significant [8].

Plants play a vital role in N and P purification in coastal wetlands. Wetland vegetation not only effectively absorbs and sequesters N and P but also regulates their migration and transformation in the soil through a variety of ecological and biochemical mechanisms. Specifically, plants take up N and P to support their growth, while the decomposition of plant litter releases nutrients back into the soil, further influencing soil physicochemical properties [9]. In the Yellow River Delta coastal wetlands, *Suaeda salsa* and *Phragmites australis* are two important dominant species that play key roles in N and P uptake and retention. *Suaeda salsa* is an annual halophyte in Amaranthaceae family characterized by high salt tolerance during seed germination and seedling stages. *Phragmites australis*, as a perennial grass in Poaceae family, has an extensive rhizome system that facilitates nutrient acquisition and storage. Numerous studies and global-scale experiments have revealed systematic differences in nutrient uptake and utilization strategies between annual and perennial species: annuals tend to adopt a “fast uptake–low resorption (nutrient resorption efficiency)–high turnover” strategy to rapidly exploit pulsed resources, whereas perennials follow a “slow uptake–high resorption–long residence” strategy to enhance nutrient retention and sustain productivity [10,11]. Moreover, a factorial experiment conducted at 94 sites across six continents demonstrated that nutrient enrichment favors grasses while disadvantaging forbs [12].

N and P are essential nutrients for plant growth and development, and their availability can significantly influence seed germination. Appropriate concentrations and forms of nutrient fertilizer to maternal plants have been shown to affect germination outcomes of offsprings. For instance, the application of N, P, and K fertilizer has been reported to promote nutrient storage in *Elymus nutans* seeds, increase seed size, and subsequently promote both seed germination and seedling growth [13]. The effects of different N forms on seed germination, with mixed N exerting greater stimulatory effects than single N forms [14]. Nitrate application has also been shown to mitigate the inhibitory effects of abscisic acid (ABA) signaling on the germination of *Capsella bursa-pastoris* seeds [15]. However, the effects of N fertilization on progeny seed germination are not consistently positive. A field fertilization experiment conducted in coastal wetlands showed that N addition significantly increased mean germination time and decreased cumulative germination percentage [16]. Such variability may result from the interactive effects of multiple factors, including species identity, soil properties, and nutrient status. However, the link between P nutrition and the germination of plant progeny seeds remains poorly understood. With the intensification of atmospheric N deposition, the N:P ratio in ecosystems continues to rise [3], and P limitation is increasingly recognized as an important factor influencing plant growth and offspring traits. Although some studies have examined the effects of N and/or P addition on seed traits in crops (e.g., nutrient content and germination) [17,18], research on how varying N:P input ratios shape offspring seed characteristics in coastal wetland plant communities remains scarce.

Seed germination is a critical stage in the plant life cycle and plays a fundamental role in plant community succession and stability. It involves two main phases: initiation, marked by chromatin remodeling and reactivation of metabolic and transcriptional activities, representing an irreversible shift from dormancy to germination competence; and visible germination, encompassing radicle protrusion, root establishment, and early seedling growth [19]. Seeds remain highly sensitive to environmental factors such as salinity [20], moisture [21], and nutrient availability [13]. For instance, moderate salt stress can promote the irreversible transition from dormancy to germination in salt-tolerant rapeseed seeds by removing repressive histone marks on *GA*-related genes via the H_2_O_2_–REF6 signaling pathway [22]. However, excessive salinity typically delays radicle protrusion and elongation, induces secondary dormancy, or even completely inhibits germination [23]. Although most current studies on coastal wetland plant seed germination focus primarily on responses to direct salt stress [16,24], the effects of long-term nutrient enrichment on the salt tolerance of progeny seeds in dominant wetland species remain poorly understood. Furthermore, whether there is a direct link between seed nutrient content and germination performance under nutrient enrichment also remains unclear.

To address these knowledge gaps, the present study employed a decade-long, multi-level, multi-ratio N and P fertilization platform in the Yellow River Delta to investigate how long-term nutrient enrichment and variations in nutrient supply ratios influence the nutrient contents, germination characteristics, and early seedling growth of the progeny seeds of dominant wetland species. Accordingly, we conducted a germination experiment using seeds of *P. australis* and *S. salsa* collected from a long-term N and P input platform in the Yellow River Delta. The specific objectives of this study were to address: (i) Does long-term N and P input alter nutrient accumulation in the seeds of *P. australis* and *S. salsa*? (ii) How do long-term nutrient inputs affect seed germination and early seedling growth under salt stress? (iii) Are seed nutrient contents significantly correlated with seed germination and early seedling growth for both species?

## 2. Results

### 2.1. Effects of Long-Term Nutrient Input on Progeny Seed N and P Contents

**Figure 1:**
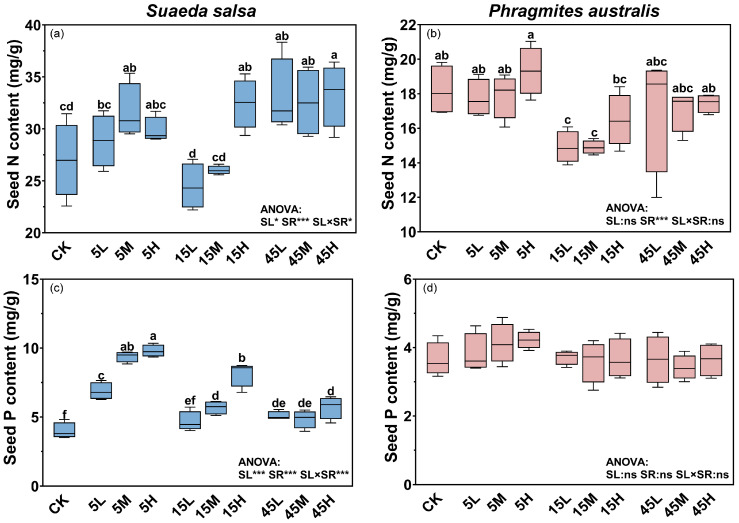
Effects of long-term fertilization on N and P contents in seeds of *S. salsa* (**a**,**c**) and *P. australis* (**b**,**d**). Different letters indicate significant differences between fertilization treatments at the 0.05 level. Asterisks indicate significant differences between different supply levels, N:P ratios or their interactions (* *p* < 0.05, *** *p* < 0.001, ns: not significant).

The nutrient contents of *S. salsa* and *P. australis* seeds exhibited distinct responses to long term N and P supply. For *S. salsa*, N and P supply levels, supply ratios, and their interaction had significant effects on seed N and P contents (*p* < 0.05, Figure 1a,c). Seed nutrient contents increased significantly with higher supply levels; compared with no-fertilization treatment (CK), N and P contents in seeds from high supply plots significantly increased by 18.0% and 98.1%, respectively (*p* < 0.05, Figure 1a,c). Seeds from plots with a high N:P ratio (45:1) generally had higher N contents (Figure 1a), whereas those from plots with a low ratio (5:1) had the highest P contents (Figure 1c). Seed N content was less responsive to fertilization, showing significant increases only under some treatments (5M, 15H, 45L, 45M, and 45H) with a range of 17.1–23.3% compared with the CK (Figure 1a, Table 1). In contrast, seed P content responded more strongly, increasing by 17.1–146.0% across fertilized plots (Figure 1c). For *P. australis*, the N and P content in seeds remained relatively stable across treatments, with only N:P supply ratio significantly influenced N content (*p* < 0.05, Figure 1b,d). Seeds from plots with medium N:P ratio (15:1) treatment had lower N contents, compared with 5:1 and 45:1 ratio treatments (Figure 1b).

### 2.2. Effects of Long-Term Nutrient Input on Progeny Seed Germination Under Different Salinity

**Figure 2:**
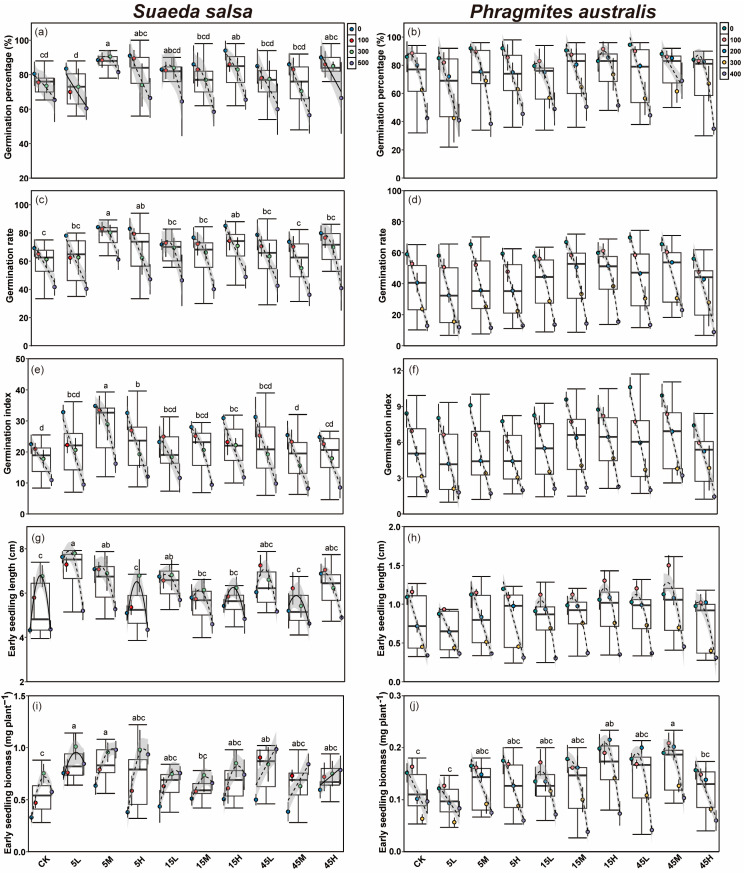
Effects of long-term fertilization and salt concentration on germination percentage (**a**,**b**), germination rate (**c**,**d**), germination index (**e**,**f**), early seedling length (**g**,**h**) and biomass (**i**,**j**) of *S. salsa* and *P. australis*. Different letters indicate significant differences between fertilization treatments at the 0.05 level. ANCOVA was used to assess differences in the slopes of regression lines among salinity treatments (Mean ± SD) within the same fertilization level. When no significant difference was detected (*p* > 0.05), the fitted line was plotted as a solid line. When ANCOVA indicated a significant difference (*p* < 0.05), the fitted line was plotted as a dashed line, with the 95% confidence interval displayed.

Salt concentration had a significant effect on the germination percentage (G), germination rate (GR) and germination index (GI) of *S. salsa* and *P. australis* seeds, with all traits showing a significant decline as salinity increased (*p* < 0.05, Table 2, Figure 2a–f). Seeds of *S. salsa* collected from long-term fertilized plots exhibited significantly improved germination traits (G, GR and GI) compared with the CK, with the highest G and GR occurring under high nutrient supply level, followed by the medium supply level (Figure 2a,c,e and Figure S1a). In contrast, *P. australis* seeds from the medium supply plots exhibited significantly higher germination traits than those from other supply levels, while seeds under low and high supply levels showed no significant differences from the CK (Figure 2b,d,f and Figure S1b). The N:P supply ratio also had a significant effect on the germination traits of both *S. salsa* and *P. australis* (*p* < 0.05, Table 2). Seeds of *S. salsa* collected from all three N:P ratios performed significantly better than the CK, with the low N:P ratio (5:1) producing the most significant enhancement. In contrast, seeds under the high N:P ratio (45:1) displayed poorer germination performance than other ratios (*p* < 0.05, Figure S1c). By contrast, *P. australis* seeds exhibited the best germination performance under medium (15:1) and high (45:1) N:P ratios, with GR and GI significantly higher than those of the CK, whereas seeds under the 5:1 ratio showed no significant difference from the CK (*p* < 0.05, Figure S1d). Furthermore, significant interactive effects among nutrient supply level, ratio, and salt concentration were observed for seed germination traits of both species (*p* < 0.05, Table 2). Specifically, the interaction between supply level and salt concentration had no significant effect on the germination traits of *S. salsa* but significantly influenced the G and GI of *P. australis* (Table 2). Similarly, the interaction between N:P ratio and salt concentration had no significant effect on *S. salsa*, whereas *P. australis* seeds showed significant responses in GR and GI (Table 2). The interaction of supply level, ratio, and salt concentration had a significant effect on the GI of *S. salsa* and G and GI of *P. australis* (*p* < 0.05, Table 2).

Recovery germination percentage of *P. australis* seeds also varied significantly across nutrient treatments, with the highest recovery (52.6%) observed in the 15H treatment and the lowest (19.5%) in 5M (*p* < 0.05, Figure S2). Notably, the 5:1 ratio led to significantly lower recovery germination compared to the other nutrient ratios (*p* < 0.05, Figure S2).

### 2.3. Effects of Long-Term Nutrient Input on the Early Seedling Growth of Progeny Under Different Salinity

Salt concentration exerted significant effects on the early seedling growth (seedling length and biomass) of *S. salsa* and *P. australis* (*p* < 0.05, Table 2, Figure 2g–j). For *S. salsa*, early seedling length at 100 and 300 mM NaCl was significantly higher than that of the control (0 mM), but declined significantly when the salt concentration reached 500 mM (*p* < 0.05, Figure 2g). The biomass of *S. salsa* early seedlings, however, increased with rising salt concentration and was significantly greater at 300 and 500 mM compared with the 0 mM (Figure 2i). In contrast, *P. australis* seedling length decreased with increasing salt concentration, and salt stress lower than 100 mM NaCl were more favorable for biomass accumulation (Figure 2h,j).

Seeds of *S. salsa* collected from long-term fertilized plots exhibited superior early growth performance after germination compared with those from unfertilized plots, while early seedlings of *P. australis* from fertilized plots also showed higher biomass relative to the CK (Figure 2g–j). Growth performance of *S. salsa* and *P. australis* early seedlings differed among nutrient supply levels (*p* < 0.05, Table 2). Under low nutrient treatment, *S. salsa* seedlings had significantly greater seedling length than those at other supply levels, while both medium and high supply also resulted in seedling lengths significantly higher than the CK, with no significant difference between them (*p* < 0.05, Figure S1a). Biomass of *S. salsa* early seedlings showed no significant differences among supply levels, but remained significantly higher than the CK across all treatments (Figure S1a). For *P. australis*, seedling length was significantly greater under medium nutrient supply compared with other nutrient levels, followed by the high supply, while that under low supply did not differ significantly from the CK (Figure S1b). Early seedling biomass of *P. australis* was significantly higher under medium and high supply than under low nutrient supply and the CK (*p* < 0.05, Figure S1b). The N:P supply ratio also significantly influenced early seedling growth of both species (*p* < 0.05, Table 2). *S. salsa* seedlings exhibited significantly better growth under 5:1 ratio compared with 15:1, 45:1 ratios, and the CK. Among the three ratios, early seedling growth was lowest under 15:1, although it was still significantly higher than the CK (*p* < 0.05, Figure S1c). In contrast, *P. australis* seedlings showed significant increases in both seedling length and biomass under 15:1 and 45:1 ratios, while no significant difference from the CK was observed under the 5:1 ratio (Figure S1d). Furthermore, supply level and supply ratio had significant interactive effects on the early seedling growth of both species (*p* < 0.05, Table 2). The interaction between supply level and salt concentration had no significant effect on the early growth of *S. salsa*, but significantly affected *P. australis* seedlings (Table 2). The interaction between supply ratio and salt concentration significantly influenced seedling length and biomass of both species (*p* < 0.05, Table 2). Moreover, the combined interaction of supply ratio, supply level, and salt concentration had significant effects on early seedling growth of *S. salsa* and *P. australis* (*p* < 0.05, Table 2).

### 2.4. Relationships Between Seed Nutrient Contents and Germination and Growth Traits Under Different Treatments

**Figure 3:**
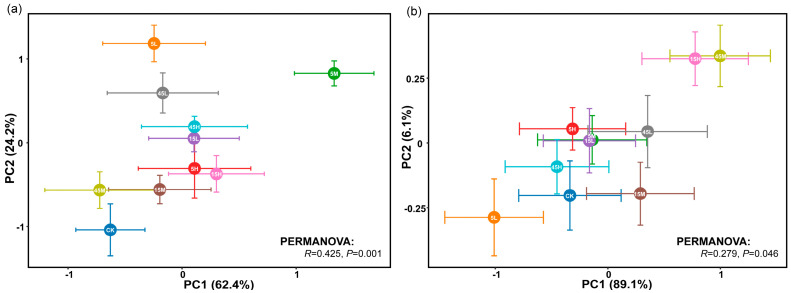
Principal component analysis (PCA) of seed germination traits and early seedling growth of *S. salsa* (**a**) and *P. australis* (**b**) under different long-term fertilization treatments. Differences in germination traits between fertilization treatments were evaluated using PERMANOVA. Statistical significance was determined at *p* < 0.05.

**Figure 4:**
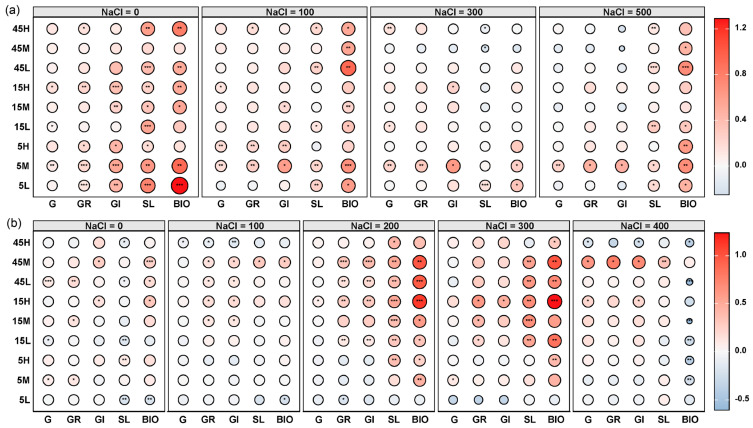
Relative effects of long-term fertilization treatments on seed germination traits and early seedling growth of *S. salsa* (**a**) and *P. australis* (**b**) under different salinity levels. Traits include germination percentage (G), germination rate (GR), germination index (GI), early seedling length (SL), and early seedling biomass (BIO). Asterisks indicate significant differences (* *p* < 0.05, ** *p* < 0.01, *** *p* < 0.001) in relative effects compared to the non-fertilized control (CK). The color scale represents the direction and magnitude of the relative effect, with circle size indicating the effect’s strength.

Principal component analysis (PCA) revealed significant differences in the germination traits and early seedling growth of *S. salsa* and *P. australis* among seeds subjected to different long-term fertilization treatments (*p* < 0.05, Figure 3). Permutational multivariate analysis of variance (PERMANOVA) further indicated that *S. salsa* seeds exhibited greater variability across treatments (*R* = 0.425, *p* = 0.001) compared with *P. australis* seeds (*R* = 0.279, *p* = 0.046), suggesting higher sensitivity of *S. salsa* to long-term fertilization disturbances (Figure 3). In most fertilization treatments, *S. salsa* seeds deviated significantly from the CK, with the largest displacement observed in the 5M treatment, indicating the strongest response, followed by 5L and 45L (Figure 3a). The relative effects of long-term fertilization generally resulted in significant positive impacts on germination and early seedling growth of *S. salsa*, especially at 0 mM NaCl, whereas the positive effect decreased as salinity increased. Notably, the 5M treatment exhibited the most significant positive response (Figure 4a). By contrast, *P. australis* seeds were more tightly clustered in the PCA, with substantial overlap among treatments and proximity to CK, indicating a relatively weak response to fertilization (Figure 3b). Fertilization effects on *P. australis* seeds were dual: seeds from the 5L treatment were inhibited, whereas those from 15H and 45M treatments showed the most significant positive responses in germination and early growth (Figure 4b).

**Figure 5:**
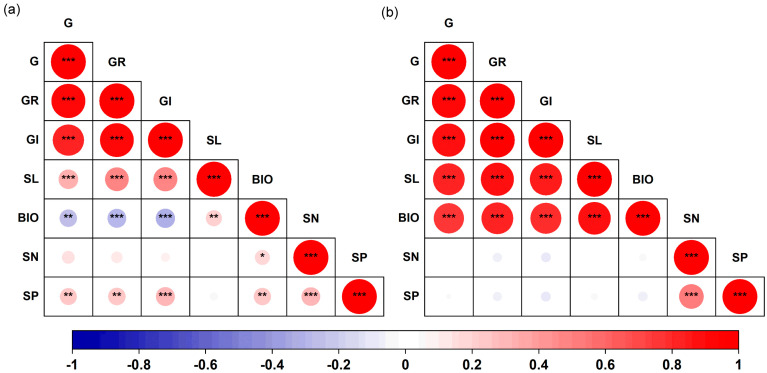
Correlation matrix for seed germination traits, early seedling growth and seed nutrient content for *S. salsa* (**a**) and *P. australis* (**b**). Note: G, germination percentage; GR, germination rate; GI, germination index; SL, early seedling length; BIO, early seedling biomass; SN, seed N content; SP, seed P content. * *p* < 0.05, ** *p* < 0.01, *** *p* < 0.001.

There were significant correlations among the germination traits of both *S. salsa* and *P. australis* (*p* < 0.05, Figure 5). In *S. salsa*, G, GR, and GI showed significant negative correlations with early seedling biomass, but significantly positively correlated with seedling length (*p* < 0.05, Figure 5a). The N content of *S. salsa* seeds was significantly positively correlated with early seedling biomass (*R* = 0.18, *p* < 0.05). The P content of the seeds was significantly positively correlated with G (*R* = 0.24, *p* < 0.01), GR (*R* = 0.26, *p* < 0.01), GI (*R* = 0.29, *p* < 0.001), biomass (*R* = 0.24, *p* < 0.01), and N content (*R* = 0.29, *p* < 0.001). In *P. australis*, significant positive correlations were observed among all germination traits (*p* < 0.001, Figure 5b). However, no significant correlations were found between seed N or P content and germination traits (Figure 5).

## 3. Discussion

Our results demonstrated that long-term N and P fertilization significantly increased N and P contents in *S. salsa* seeds, particularly under high nutrient supply, whereas *P. australis* seeds maintained relatively stable nutrient levels. As an annual halophyte with a short life cycle, *S. salsa* is highly sensitive to environmental changes and relies primarily on seed reproduction. Therefore, under nutrient-enriched conditions, *S. salsa* may enhance offspring fitness by increasing seed nutrient reserves. In contrast, *P. australis*, a perennial clonal grass, primarily propagates vegetatively, although it can also reproduce via seeds. This disparity may stem from functional group-specific ecological strategies in nutrient acquisition and use efficiency. Numerous studies have shown that fertilization, especially N addition, can alter plant community composition, with grasses typically exhibiting weaker responses than annual or biennial herbs and legumes [12,25,26]. A study on *Leymus chinensis*, which was also a perennial clonal grass, found that N addition enhanced carbon and nitrogen translocation from reproductive to vegetative ramets [27]. Similarly, under nutrient-enriched conditions, *P. australis* may preferentially allocate excess resources to rhizomes, thereby maintaining relatively stable seed nutrient composition. This allocation pattern is likely linked to its clonal integration strategy, through which interconnected ramets can share water, nutrients, and photosynthates [28]. Such physiological integration allows the clone to buffer local environmental heterogeneity, stabilizing nutrient status and reducing the sensitivity of reproductive structures to external nutrient fluctuations. Consequently, while this conservative allocation strategy may constrain rapid seed-based expansion in nutrient-rich environments, it enhances long-term population persistence and resilience under fluctuating coastal conditions. Although the nutrient content in *S. salsa* seeds was significantly affected by long-term fertilization, N content remained relatively stable compared to P, consistent with previous findings suggesting that N in plant tissues is generally more stable than P. This is because N uptake is mainly regulated by biotic factors and N is largely accumulated in proteins and nucleic acids, whereas P is a more labile element, primarily stored in membranes as phospholipids, with its availability influenced by both biotic and abiotic processes [29,30].

Seed N and P reserves are critical determinants of germination, synergistically regulating this process by modulating key enzymatic activities, coordinating metabolic pathways, and maintaining adequate energy supply [31]. Correlation analysis showed that P contents of *S. salsa* seed significantly positively correlated with germination parameters and early seedling biomass, while N content only significantly promoted early seedling biomass accumulation. In contrast, no significant correlations were found between N and P contents and seed germination or early seedling growth in *P. australis*. These findings suggest that *S. salsa* seed germination relies more heavily on internal nutrient reserves than *P. australis* does. HrdliČKovÁ et al. [32] demonstrated that the availability and content of P in Rumex obtusifolius seeds are critical factors influencing germination behavior. Phosphorus in seeds is primarily stored as inositol hexakisphosphate (InsP_6_), which undergoes hydrolysis during germination to release inorganic P and essential mineral elements, thereby supporting early seedling growth [33]. As an annual plant, *S. salsa* must complete its life cycle within a single growing season. Salt stress significantly reduces soil P availability [34]; therefore, when P availability is higher, maternal plants may increase P allocation to seeds, representing a potential transgenerational adaptive strategy. In this way, the maternal plant can anticipate the potential environmental challenges faced by offspring based on its own growth conditions and convert resources into offspring pre-adaptive advantages through non-genetic mechanisms. Such strategies are particularly common in annual and pioneer species, enhancing early offspring performance under stressful conditions [35]. The direct link between maternal P environment and offspring performance enables *S. salsa* to establish more rapidly and gain a competitive advantage in nutrient-variable and frequently disturbed coastal wetlands.

Our results indicated that long-term N and P inputs of maternal environments significantly affected the seed germination and early seedling growth of *S. salsa* and *P. australis*, with *S. salsa* showing greater sensitivity to different fertilization regimes. This suggests that sustained external N and P inputs not only significantly influence maternal nutrient status [36], but also affect seed germination performance and early seedling establishment. Moreover, plant functional groups in coastal wetlands exhibit different strategies in response to long-term nutrient enrichment. In our study, seeds from *S. salsa* and *P. australis* communities exhibited distinct responses to varying levels of N and P supply. *S. salsa*, as an annual species, showed higher sensitivity in seed responses to fertilization and served as a strong indicator of nutrient perturbation. In contrast, the perennial grass *P. australis*, with its well-developed storage organs and ecological buffering mechanisms, exhibited relatively limited changes in seed germination and early seedling growth under long-term fertilization. Previous studies have shown that in forest ecosystems, low to medium levels of N or P input can promote seed germination in multiple species, whereas high concentrations may exert toxic effects [26,37]. However, the sensitivity of seed germination to nutrient input is species-dependent [38]. Our findings indicated that long-term high levels of N and P supply significantly enhanced nutrient accumulation in *S. salsa* seeds, thereby increasing internal nutrient reserves and promoting seed germination. For *P. australis* seeds, medium supply levels were more favorable for germination. Although prolonged external nutrient input did not significantly alter the nutrient reserves in the seeds, it may have influenced germination by modulating hormone biosynthesis pathways during seed development. Previous studies have reported that appropriate N input can promote seed germination by reducing the ratio of abscisic acid (ABA) to gibberellins (GA) [39,40]. In this study, long-term N:P supply ratio also significantly influenced germination and growth performance of *S. salsa* and *P. australis*. For *S. salsa*, the low N:P ratio treatment (5:1) was most effective in promoting seed germination and early seedling growth. This finding supports our inference that high P availability enhances seed P accumulation, which is strongly positively correlated with seed germination and seedling biomass. In contrast, *P. australis* exhibited enhanced germination and seedling growth under higher N:P ratios (15:1 and 45:1). Compared with P, the availability of N more effectively promoted the germination and early seedling growth performance of *P. australis*. This may be attributed to the maternal-stage response of *P. australis* to N input, which increases aboveground biomass and chlorophyll synthesis, thereby enhancing photosynthetic capacity and indirectly improving seed development quality [41]. These species-specific responses to nutrient levels and N:P ratios may reflect distinct nutrient-use strategies evolved under variable environmental conditions and are likely to shape differential adaptation potentials under future climate scenarios.

In coastal wetlands, salt is a major factor influencing seed germination and early seedling establishment [16]. In this study, the progeny seed germination of both species decreased with increasing salt stress, however, the biomass of *S. salsa* early seedlings significantly increased, whereas *P. australis* seedlings exhibited the greatest biomass under low salinity, which is associated with the differing salt tolerance of the two species. As a halophyte, *S. salsa* can exclude, accumulate, and absorb salt, enabling it to thrive under high-salinity conditions [23]. Although *P. australis* possesses some degree of salt tolerance, high salinity can cause ion toxicity to the plant [42]. Previous study have proved that seed dormancy enable plants to evade high salt stress and resume germination once favorable conditions occur, but prolonged or severe salt stress can result in a permanent loss of seed viability, reducing their recovery potential [43]. Our study revealed significant variation in the post-stress germination capacity of *P. australis* seeds from different nutrient treatments, with seeds from higher N:P ratio treatments (15:1 and 45:1) displaying enhanced recovery potential. This suggests that long-term elevated N inputs may enhance seed quality and stress resilience in *P. australis*, thereby improving the ability to resume growth after salt stress is relieved. Furthermore, our results also identified significant interactive effects between long-term N and P fertilization and salt stress on seed germination and early seedling growth. Notably, increasing salinity diminished the beneficial effects of fertilization on *S. salsa* seeds. While long-term fertilization improves nutrient accumulation and germination vigor in *S. salsa* seeds, high salinity may inhibit germination by restricting water uptake and endosperm hydrolysis due to osmotic imbalance and ion toxicity [44,45]. Even when seeds possess substantial internal nutrient reserves, these resources may not be fully mobilized or utilized under high salinity, effectively functioning as a “sunk cost.” This environment-dependent trade-off indicates that maternal nutrient investment can significantly enhance progeny fitness under low stress, but its advantages are constrained under high salinity. Furthermore, these findings underscore the importance of considering abiotic stress when evaluating the reproductive strategies and ecological performance of coastal wetland plants under nutrient-enriched conditions and provide a more comprehensive understanding of the interactions between maternal environment, seed nutrient status, and offspring ecological adaptation.

## 4. Materials and Methods

### 4.1. Study Materials

The seeds of *P. australis* and *S. salsa* were collected from the long-term nutrient input platform at the Yellow River Delta Research Station, Chinese Academy of Sciences (37°45′52″ N, 118°58′52″ E). The nutrient input platform was established in 2014, comprising 3.5 m × 3.5 m fenced plots with relatively homogeneous vegetation, predominantly composed of natural mixed *P. australis* and *S. salsa* communities. A fully factorial experimental design was employed, combining three N:P supply ratios (5:1, 15:1, and 45:1) with three nutrient supply levels—low (L), medium (M), and high (H)—to represent N-limited, balanced, and P-limited nutrient conditions, respectively [46]. An unfertilized control (CK) was included, and each treatment was replicated four times (Table 1). At each N:P ratio, N supply was increased in threefold increments to establish a continuous nutrient gradient. For each treatment, the overall nutrient supply (L) was calculated as the geometric mean of N and P additions [47], as follows:

$$N\left(g\right)=L\left(g\right)\cdot \sqrt{N:P}$$

$$P\left(g\right)=L(g)/\sqrt{N:P}$$

where N and P represent the annual additions of N and P (g m^−2^ y^−1^). This approach allowed assessment of N:P ratio effects independently from differences in total nutrient input.

The baseline N addition under the 15:1 ratio (basic N and P supply) was set at 5 g m^−2^ y^−1^, based on atmospheric deposition in the growing seasons (2.26 g m^−2^) and background inputs in the Yellow River Delta [48]. Fertilization (N: urea; P: NaH_2_PO_4_) began in 2015 and was applied twice annually—in early April during the sprouting stage and in late June during the peak growth period [49]. Fertilizer application continued for 10 consecutive years until 2024. Seeds of *P. australis* and *S. salsa* were collected from the experimental plots during their respective maturity stage in November (*P. australis*) and December (*S. salsa*) 2024. After collection, seeds were stored at 4 °C until the germination experiments were conducted in early spring 2025.

### 4.2. Measurements of Seed N and P Contents

Seeds of *S. salsa* and *P. australis* were oven-dried at 60 °C until a constant weight was achieved, and 100 mg dry seeds of *S. salsa* and *P. australis* were weighted and finely ground for N and P content analysis. Seed N content was determined using the Dumas dry com-bustion method with an elemental analyzer [50]. Approximately 20–30 mg of ground seeds was weighed, placed into a tin capsule, and combusted at ~950 °C in pure oxygen. N was converted to N_2_, quantified using a thermal conductivity detector (TCD), and calibrated with acetanilide. Seed P content was determined using the molybdenum–antimony anti-colorimetric method with an automated flow injection analyzer [50]. About 50 mg of ground sample was digested in 8 mL concentrated sulfuric acid for 12 h, followed by heating with hydrogen peroxide until the solution turned light blue. The digest was cooled, diluted to 50 mL, and 1 mL aliquots were mixed with 2 mL distilled water, incubated at 45 °C for 25 min, and measured at 660 nm using a spectrophotometer. P concentration was calculated based on a standard curve with a blank as control.

### 4.3. Seed Germination Experiments

Based on previous experimental validation and the known salinity tolerance ranges of the two species in their natural habitats [51,52], NaCl solutions at concentrations of 0, 100, 300, and 500 mM were used to simulate salt stress during germination of *S. salsa* seeds, while five salinity levels (0, 100, 200, 300, and 400 mM) were applied for *P. australis*. The experiment was conducted using a 1% agar medium, with 20 mL of NaCl-amended agar solution poured into each Petri dish. After solidification, 50 healthy seeds were evenly placed on the surface, with four replicates per treatment, totaling 200 seeds per treatment. Prior to plating, seeds were surface-sterilized with 3% hydrogen peroxide for 15 min and then thoroughly rinsed with distilled water. To prevent moisture loss, the dishes were sealed with parafilm and incubated in a growth chamber under a 12 h light/12 h dark photoperiod at a light intensity of 22 μmol/m^2^/s at alternating temperature of 25/15 °C (light/dark). Germination was defined as seedling emergence of at least 1 mm, and germinated seeds were counted every 24 h. The duration of germination experiment was 10 days for *S. salsa* and 15 days for *P. australis*, terminating after three consecutive days with no additional germination. At the end of the experiment, a large number of *P. australis* seeds remained ungerminated under the 400 mM salinity treatment. To understand the tolerance of *P. australis* seeds in high salinity habitat, the ungerminated seeds were thoroughly rinsed with distilled water and transferred to Petri dishes lined with double-layered filter paper with 10 mL distilled water for a recovery germination test.

### 4.4. Measurement Indexes and Methods

Germination and early seedling growth related traits including germination percentage, germination rate, germination index, recovery percentage, seedling length, and seedling biomass, were measured and calculated as follows.

Germination percentage (G, %) was calculated as:

G = (Final number of germinated seeds/Total number of seeds tested) × 100%.

Germination rate (GR) was calculated as:

GR = Σ(G/t), where G is the daily germination percentage and t is the corresponding germination day [53].

Germination index (GI) was calculated as:

GI = Σ(G_t_/D_t_), where G_t_ is the number of seeds germinated on day t, and D_t_ is the corresponding day of germination.

Recovery percentage (RP) was calculated as:

RP = (Number of seeds germinated during the recovery test/Number of ungerminated seeds after the germination test) × 100%.

After the germination experiment, five *S. salsa* or *P. australis* early seedlings were randomly selected from each Petri dish to measure early seedling length with 0.1 cm accuracy [54]. For salt-treated groups, excess surface salts were thoroughly removed by rinsing the seedlings with distilled water. All seedlings were then oven-dried at 60 °C for 48 h, and dry weight was determined using an analytical balance with a precision of 0.0001 g.

### 4.5. Statistical Analyses

All datasets were tested for normality prior to statistical analysis. Non-normally distributed datasets were transformed prior to analysis. All statistical analyses were performed using SPSS 26.0. One-way ANOVA and general linear models (GLMs) were used to assess the effects of salinity, nutrient supply levels, and N:P ratios on G, GR, GI, recovery percentage, early seedling length and biomass. Three-way ANOVA was used to test the main and interactive effects of species, nutrient supply level, and N:P ratio on G, GR, GI, early seedling length and biomass. In addition, three-way ANOVA was separately performed to assess the effects of salinity, nutrient supply level, and N:P ratio on *S. salsa* and *P. australis* seeds. A two-way ANOVA was used to evaluate the main effects and interactions of supply level and supply ratio on seed nutrient content and recovery percentage. Tukey’s HSD post hoc test was conducted to identify significant pairwise differences among treatments. Statistical significance was determined at *p* < 0.05. Regression slopes between the response variables (G, GR, GI, early seedling length and biomass) and salinity were estimated using analysis of covariance (ANCOVA), with statistical significance determined at *p* < 0.05. PCA was conducted using the “factoextra,” “FactoMineR,” and “ggplot2” packages in R version 4.1.3. Based on Euclidean distances, PERMANOVA was performed to assess the significance of multivariate differences among treatment groups.

To evaluate the influence of long-term fertilization on the seed germination and early seedling growth of *S. salsa* and *P. australis*, relative effects (REs) were calculated using the following formula:

$$RE=\frac{Treatment-Control}{Control}$$

In this analysis, each treatment value was treated as an individual observation, while the control value was represented by the mean of four replicate control plots. A positive RE value indicates an increase relative to the unfertilized control, whereas a negative RE value indicates a decrease. To determine whether fertilization treatments differed significantly from the control, a *t*-test was conducted.

## 5. Conclusions

By testing effects of long-term N and P input on seed nutrient contents, seed germination and early seedling growth of typical wetland plants, distinct response patterns were observed between the two dominant species, *S. salsa* and *P. australis*. In terms of seed nutrient content, long-term fertilization significantly increased nutrient content in *S. salsa* seeds, with seed P content showing significant positive correlations with G, GR, GI, and seedling biomass. In contrast, the N and P contents in *P. australis* seeds remained relatively stable across different fertilization treatments and exhibited insignificant correlations with germination and seedling growth. Long-term N and P inputs generally had positive effects on seed germination and early seedling growth of *S. salsa*, especially under high nutrient supply or a 5:1 supply ratio. In contrast, germination and growth performance of *P. australis* seeds significantly improved under medium nutrient supply level or higher N:P ratios (15:1 and 45:1). *S. salsa* seeds exhibited relatively more sensitive and variable germination and growth responses to long-term fertilization compared to *P. australis*. These findings offer a foundation for forecasting plant community dynamics and informing the management of coastal wetlands.

## Figures and Tables

**Table 1:** Nitrogen and phosphorus input amounts under different nutrient addition treatments.

Supply Ratio	Supply Level	Treatment	N Input Amount (g/m^2^·Year)	P Input Amount (g/m^2^·Year)
5:1	Low	5L	2.89	0.58
	Medium	5M	8.67	1.73
	High	5H	26.01	4.19
15:1	Low	15L	5.00	0.33
	Medium	15M	15.00	1.00
	High	15H	45.00	3.00
45:1	Low	45L	8.67	0.19
	Medium	45M	26.01	0.58
	High	45H	78.03	1.73

**Table 2:** Three-way ANOVA results showing the effects of nitrogen and phosphorus supply level (SL), supply ratio (SR), and salt concentration (SC), and their interactions on seed germination percentage, germination rate, germination index, early seedling length and biomass of *S. salsa* and *P. australis*. Asterisks indicate significance levels (* *p* < 0.05, ** *p* < 0.01, *** *p* < 0.001).

Index	SL	SR	SC	SL × SR	SL × SC	SR × SC	SL × SR × SC
*Suaeda salsa*							
Germination percentage	8.737 ***	1.223	38.007 ***	6.021 ***	1.238	0.511	1.309
Germination rate	5.636 **	5.431 **	59.473 ***	6.785 ***	1.642	0.329	1.157
Germination index	3.347 *	25.172 ***	168.484 ***	11.361 ***	0.623	1.123	1.873 *
Early seedling length	42.15 ***	10.182 ***	99.256 ***	21.327 ***	0.964	5.838 ***	2.942 **
Early seedling biomass	5.758 **	27.523 ***	78.334 ***	6.539 ***	1.359	6.002 ***	3.045 **
*Phragmites australis*							
Germination percentage	10.69 ***	4.599 *	186.227* **	3.683 **	2.745 **	1.887	2.699 **
Germination rate	13.561 ***	43.661 ***	586.966 ***	10.48 ***	1.653	5.363 ***	1.323
Germination index	15.299 ***	43.056 ***	542.12 ***	12.542 ***	3.445 **	2.714 **	1.871 *
Early seedling length	27.834 ***	38.626 ***	724.398 ***	29.478 ***	7.568 ***	12.874 ***	3.542 ***
Early seedling biomass	24.787 ***	43.851 ***	246.267 ***	39.3 ***	2.105 *	8.491 ***	5.874 ***

Note: Values in bold indicate statistically significant effects.

## Data Availability

Data are contained within the article and its Supplementary Materials. Further inquiries can be directed at the corresponding author.

## Supplementary Materials

The following supporting information can be downloaded at: https://www.mdpi.com/article/10.3390/plants14213393/s1, Figure S1: Radar plots showing germination percentage (G), germination rate (GR), germination index (GI), seedling length (SL), and biomass (BIO) of *S. salsa* (a,b) and *P. australis* (c,d) under different N and P supply levels or N:P ratios. Different letters indicate significant differences between N and P supply levels or N:P ratios at the 0.05 level.; Figure S2: Effects of long-term fertilization on recovery germination percentage of *P. australis*. Different letters indicate significant differences between fertilization treatments at the 0.05 level. Asterisks indicate significant differences between different supply levels, N:P ratios or their interactions (* *p* < 0.05, ** *p* < 0.01, *** *p* < 0.001, ns: not significant).
