# Supplementary material for: Effects of Long-Term Nutrient Input on Progeny Seed Nutrient Contents, Germination and Early Growth Characteristics of Typical Coastal Wetland Plants
Source: Plants (Basel). 2025 Nov 5;14(21):3393. doi: 10.3390/plants14213393 (PMC12608885; doi:10.3390/plants14213393)
Supplement: Supplementary file 1 [file plants-14-03393-s001.zip › plants-3938336-supplementary.pdf]

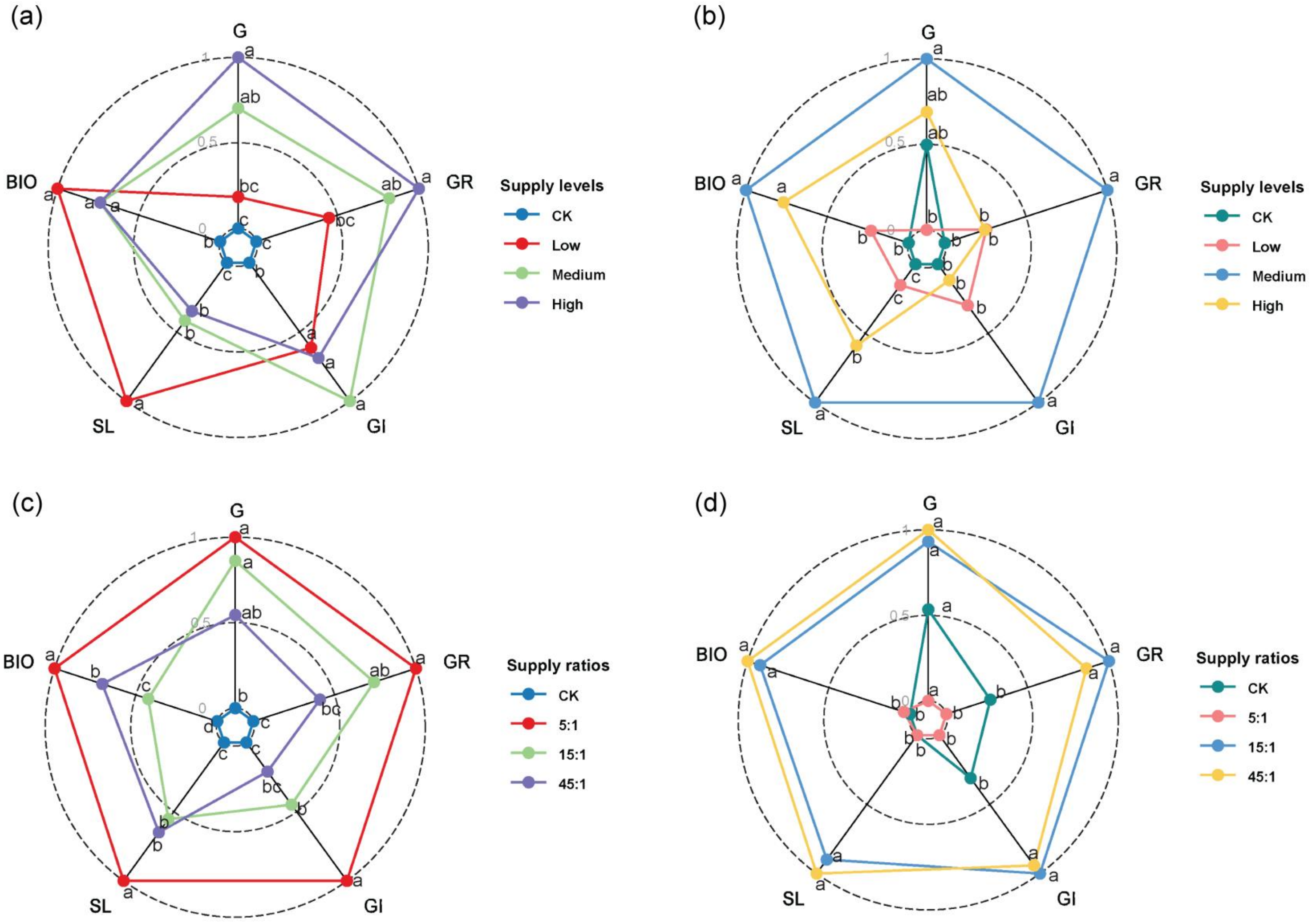

Figure S1. Radar plots showing germi
and *P. australis* (c,d)under different N
ratios at the 0.05 level.

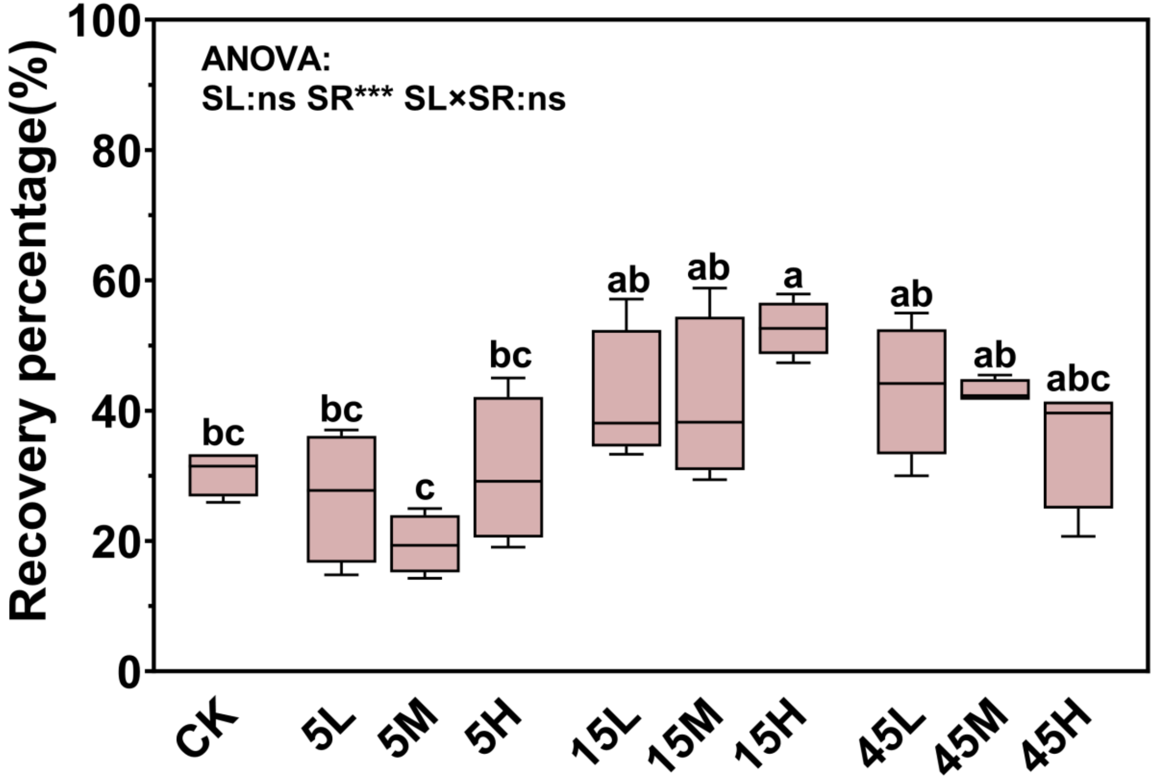

Figure S2. Effects of long-term fertilization on recovery germination percentage of *P. australis*. Different letters indicate significant differences between fertilization treatments at the 0.05 level. Asterisks indicate significant differences between different supply levels, N:P ratios or their interactions (* $p < 0.05$, ** $p < 0.01$, *** $p < 0.001$, ns: not significant).
